# Quality of Life and Physical Activity in 629 Individuals With Sarcoidosis: Prospective, Cross-sectional Study Using Smartphones (Sarcoidosis App)

**DOI:** 10.2196/38331

**Published:** 2022-08-10

**Authors:** Brian Chu, Daniel M O'Connor, Marilyn Wan, Ian Barnett, Haochang Shou, Marc Judson, Misha Rosenbach

**Affiliations:** 1 Department of Dermatology Perelman School of Medicine University of Pennsylvania Philadelphia, PA United States; 2 Department of Dermatology Harvard Medical School Boston, MA United States; 3 Department of Biostatistics, Epidemiology, and Informatics Perelman School of Medicine University of Pennsylvania Philadelphia, PA United States; 4 Division of Pulmonary and Critical Care Medicine Albany Medical College Albany, NY United States

**Keywords:** sarcoidosis, smartphone, quality of life, mobile app, mobile health, mHealth, digital health, rare disease, physical activity, exercise, fitness, development, tracking, recruit, enroll

## Abstract

**Background:**

Large gaps exist in understanding the symptomatic and functional impact of sarcoidosis, a rare multisystem granulomatous disease affecting fewer than 200,000 individuals in the United States. Smartphones could be used for prospective research, especially for rare diseases where organizing large cohorts can be challenging, given their near ubiquitous ownership and ability to track objective and subjective data with increasingly sophisticated technology.

**Objective:**

We aimed to investigate whether smartphones could assess the quality of life (QoL) and physical activity of a large cohort of individuals with sarcoidosis.

**Methods:**

We developed a mobile app (Sarcoidosis App) for a prospective, cross-sectional study on individuals with sarcoidosis. The Sarcoidosis App was made available on both Apple and Android smartphones. Individuals with sarcoidosis were recruited, consented, and enrolled entirely within the app. Surveys on sarcoidosis history, medical history, and medications were administered. Patients completed modules from the Sarcoidosis Assessment Tool, a validated patient-reported outcomes assessment of physical activity, fatigue, pain, skin symptoms, sleep, and lungs symptoms. Physical activity measured by smartphones was tracked as available.

**Results:**

From April 2018 to May 2020, the App was downloaded 2558 times, and 629 individuals enrolled (404, 64.2% female; mean age 51 years; 513, 81.6% White; 86, 13.7% Black). Two-thirds of participants had a college or graduate degree, and more than half of them reported an income greater than US $60,000. Both QoL related to physical activity (*P*<.001, ρ=0.250) and fatigue (*P*<.01, ρ=–0.203) correlated with actual smartphone-tracked physical activity. Overall, 19.0% (98/517) of participants missed at least 1 week of school or work in an observed month owing to sarcoidosis, and 44.4% (279/629) reported that finances “greatly” or “severely” affected by sarcoidosis. Furthermore, 71.2% (437/614) of participants reported taking medications for sarcoidosis, with the most common being prednisone, methotrexate, hydroxychloroquine, and infliximab. Moreover, 46.4% (244/526) reported medication side effects, most commonly due to prednisone.

**Conclusions:**

We demonstrate that smartphones can prospectively recruit, consent, and study physical activity, QoL, and medication usage in a large sarcoidosis cohort, using both passively collected objective data and qualitative surveys that did not require any in-person encounters. Our study’s limitations include the study population being weighted toward more educated and wealthier individuals, suggesting that recruitment was not representative of the full spectrum of patients with sarcoidosis in the United States. Our study provides a model for future smartphone-enabled clinical research for rare diseases and highlights key technical challenges that future research teams interested in smartphone-based research for rare diseases should anticipate.

## Introduction

Sarcoidosis is a multisystem granulomatous disease that most commonly affects the lungs, skin, eyes, and lymph nodes. In the United States, over 185,000 patients with sarcoidosis seek medical care annually, and 25,000 new cases of sarcoidosis are diagnosed [1]. While many cases appear mild, the disease can cause substantial functional morbidity including exertional dyspnea, generalized pain, and decreased physical activity [2]. Fatigue is common among patients with sarcoidosis and can be debilitating. Given the variable clinical presentation of sarcoidosis, large gaps remain in understanding the daily impact of sarcoidosis.

Sarcoidosis is a rare disease, which is defined as affecting fewer than 200,000 individuals. Clinical research of rare diseases is challenging—nearly one-third of rare disease clinical trials are discontinued with insufficient patient accrual being the most common reason [3]. Large cohorts are difficult to recruit and may not be representative of the patient population owing to overrepresentation of patients with access to academic medical centers. However, more than 85% of Americans currently own a smartphone, and manufacturers are increasingly including sophisticated health-tracking technology [4]. For example, Apple ResearchKit allows investigators to not only measure the number of daily walking steps and distance traveled, but also estimate cardiac fitness levels and walking stability [5]. In addition, mobile apps allow patients to complete validated survey instruments on smartphones. Therefore, smartphones are a compelling channel to conduct clinical research on rare disease populations.

The goal of our study was to develop and launch a smartphone app to assess patient-reported quality of life (QoL) in a large population of patients with sarcoidosis and to characterize patients’ symptomatology and functional status. We designed this app to describe physical activity and correlate physical activity data with self-reported characteristics through QoL surveys, medication use, and adverse effects and comorbidities. Here, we describe the development and launch of the app and present results from the surveys and physical activity data collected from patients.

## Methods

### Smartphone App Development and Launch

**Figure 1:**
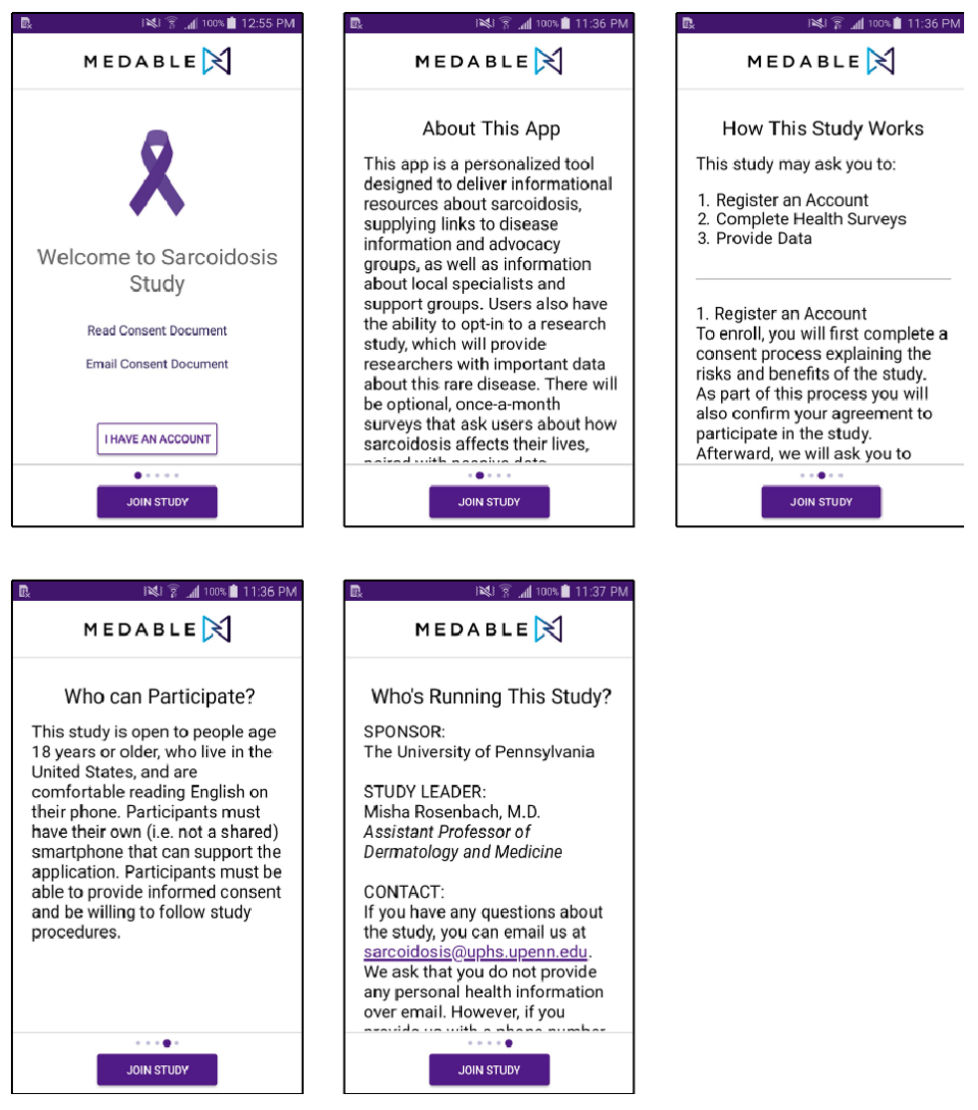
Screenshots of new participant experience in the Sarcoidosis App (Android version).

The Sarcoidosis App [6], developed by authors DMO and MR, is a smartphone app that measures physical activity levels and records patient-reported responses to questions (Figure 1) [7]. The Sarcoidosis App was initially designed using the open-source Apple ResearchKit framework. The app was then ported onto the Medable trial platform [8], to allow for distribution on both Android and Apple operating systems. Data were automatically encrypted, deidentified, and uploaded directly to secure servers, adhering to guidelines specified by the Health Insurance Portability and Accountability Act. Study data were not shared with any organization including Apple, Alphabet, Medable, or with nonstudy personnel. The Sarcoidosis App was made available on the Google Play and the Apple App Store in April 2018. Data were collected through May 2020, though the length of time spent using the App varied by participant. The Foundation for Sarcoidosis Research shared an announcement about the Sarcoidosis App’s launch with their patient email list.

### Patient Recruitment, Consent, and Enrollment

Patients with sarcoidosis were recruited via outpatient dermatology clinic visits at the University of Pennsylvania Health System, the Foundation for Sarcoidosis Research mailing list, and targeted social media advertisements. After prospective participants downloaded the app, they were presented with an inclusion and exclusion criteria questionnaire to provide multiple means of participant recruitment. Participants were eligible for study enrollment if they were aged greater than 18 years, lived in the United States, and self-reported a diagnosis of sarcoidosis. Participants were asked for permission to enable the app to read HealthKit or Google Fit data. To ensure participants understood the risks, benefits, and options of study participation, they were required to pass a quiz concerning these issues before digitally signing the informed consent document (Multimedia Appendix 1).

### Patient-Reported Outcome Measures: Survey Design and Data Collection

The Sarcoidosis App administered baseline surveys of (1) sarcoidosis history, (2) pertinent medical history, (3) sarcoidosis treatment medications, and (4) items from modules of the Sarcoidosis Assessment Tool (SAT). The SAT is a validated patient-reported assessment that comprises select generic measures from the Patient-Reported Outcomes Measurement Information System, as well as several sarcoidosis-specific item banks, including physical functioning, satisfaction with roles and activities, fatigue, pain interference, sleep disturbance, lung concerns, skin concerns, and skin stigma and embarrassment [7]. After the intake process, several surveys were administered. Participants could skip questions that they did not wish to answer and could be completed at any time. The app also asked participants for permission to import certain smartphone physical activity data, including daily counts of steps, distance walked or run, flights of stairs climbed, and exercise time.

### Statistical Analysis

Descriptive statistics were calculated for demographic information, patient-reported outcomes, and physical activity data. Missing data were excluded from analyses except where described. Spearman correlation coefficients were calculated for correlating responses to the SAT modules for physical activity, lungs, and fatigue with average daily steps and traveled distance from smartphone-recorded data, as much of the survey and physical activity measures were not normally distributed. In addition, the use of ranks through Spearman correlation diminishes the influence of outliers in some of the physical activity measures. All statistics were calculated using Stata (version 16.1; StataCorp) and R (version 3.6.1; The R Foundation for Statistical Computing). The survey data and physical activity data used in this study is available upon reasonable request to BC and MR.

### Ethical Considerations

This study was approved by the institutional review board of the University of Pennsylvania (824080). This paper adheres to STROBE (Strengthening the Reporting of Observational studies in Epidemiology) reporting guidelines. Participants who met eligibility criteria proceeded to provide electronic informed consent.

## Results

### Study Enrollment and Background Information

From April 2018 to May 2020, the app was downloaded 2558 times: 1603 from the Apple App Store and 955 from Google Play. A quarter (629/2558) of downloads converted to study participation, with 629 unique participants completing at least one component of the background survey concerning basic demographic data (Table 1). Of them, 64.2% (n=404) of participants were female, and 81.6% (n=513) were White. Two-thirds (n=416, 66.1%) of participants had a college or graduate degree, and more than half reported full-time employment (n=316, 50.2%) and annual incomes greater than US $60,000 (n=360, 57.2%). In terms of background information on sarcoidosis disease and QoL, 60.1% (n=378) of participants reported that their sarcoidosis was diagnosed within the past 5 years. Subjectively, 19.9% (n=125) of participants reported “poor” health and 39.7% (n=250) reported “fair” health. Overall, 44.4% (n=279) of participants reported that their family’s finances were “greatly affected” or “severely affected” by sarcoidosis.

**Table 1:** Baseline characteristics of participants in the Sarcoidosis App study (N=629).

Characteristic		Value
**Gender**		
	Female	404 (64.2)
	Male	199 (31.6)
	Missing	26 (4.1)
Mean age (years), mean (median; SD)		51.0 (50; 10.95)
**Race, n (%)**		
	White	513 (81.6)
	Black or African American	86 (13.7)
	Other/Unknown	27 (4.3)
	Missing	3 (0.5)
**Ethnicity, n (%)**		
	Hispanic/Latino	32 (5.1)
	Non-Hispanic	587 (93.3)
	Missing	10 (1.6)
BMI^a^, mean (SD)		32.12 (7.75)
**Years since diagnosis, n (%)**		
	<1	127 (20.2)
	1-5	251 (39.9)
	5-20	197 (31.3)
	>20	52 (8.3)
	Missing	2 (0.3)
**Education, n (%)**		
	High school	8 (1.3)
	General Educational Development	205 (32.6)
	College	266 (42.3)
	Graduate	150 (23.8)
**Employment status, n (%)**		
	Student	6 (1.0)
	Part-time	44 (7.0)
	Full-time	316 (50.2)
	Unemployed	61 (9.7)
	Disabled	114 (18.1)
	Retired	82 (13.0)
	Missing	6 (1.0)
**Income (US $), n (%)**		
	<15,000	49 (7.8)
	15,000-30,000	58 (9.2)
	30,000-60,000	139 (22.1)
	60,000-100,000	166 (26.4)
	>100,000	194 (30.8)
	Missing	23 (3.7)
**Financial impact, n (%)**		
	No financial impact	124 (19.7)
	Slightly affected	223 (35.5)
	Greatly affected	178 (28.3)
	Severely affected	101 (16.1)
	Missing	3 (0.5)

^a^BMI was obtained from 605 participants.

### SAT Results

In total, 597 unique participants filled out at least one of the SAT modules. The mean scores of each SAT module and their SDs are reported in Table 2. Owing to technical issues with the app, there were fewer reported outcomes for the skin symptoms module of the SAT.

**Table 2:** Sarcoidosis Assessment Test (SAT) survey results at baseline. A score of 50 represents the mean score of the original calibration sample of the SAT.

SAT module	Participants, n	Module score, mean (SD)
Activity [+]^a^	552	39.90 (7.97)
Fatigue [–]^b^	564	62.67 (9.32)
Lungs [–]	572	45.87 (8.46)
Pain [–]	544	60.48 (10.63)
Skin symptoms [–]	208	57.80 (6.99)
Sleep [–]	567	58.38 (9.51)
Stigma/embarrassment/skin impact [–]	535	49.73 (8.65)

^a^A higher score representing a higher quality of life.

^b^A higher score representing a lower quality of life.

### Correlational Data

**Figure 2:**
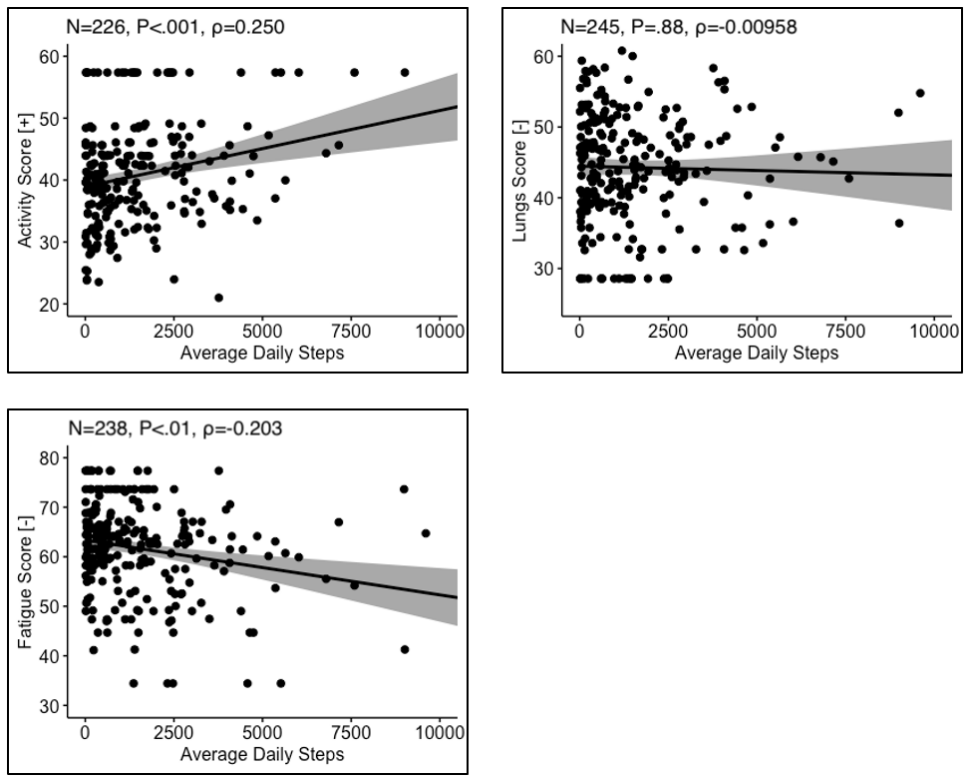
Correlation between Sarcoidosis Assessment Test surveys of physical activity, lung symptoms, and fatigue with device-reported physical activity data. [+] indicates that a higher score represents a higher quality of life. [-] indicates that a higher score represents a lower quality of life. P and ρ are the *P* value and Spearman correlation coefficient, respectively.

**Figure 3:**
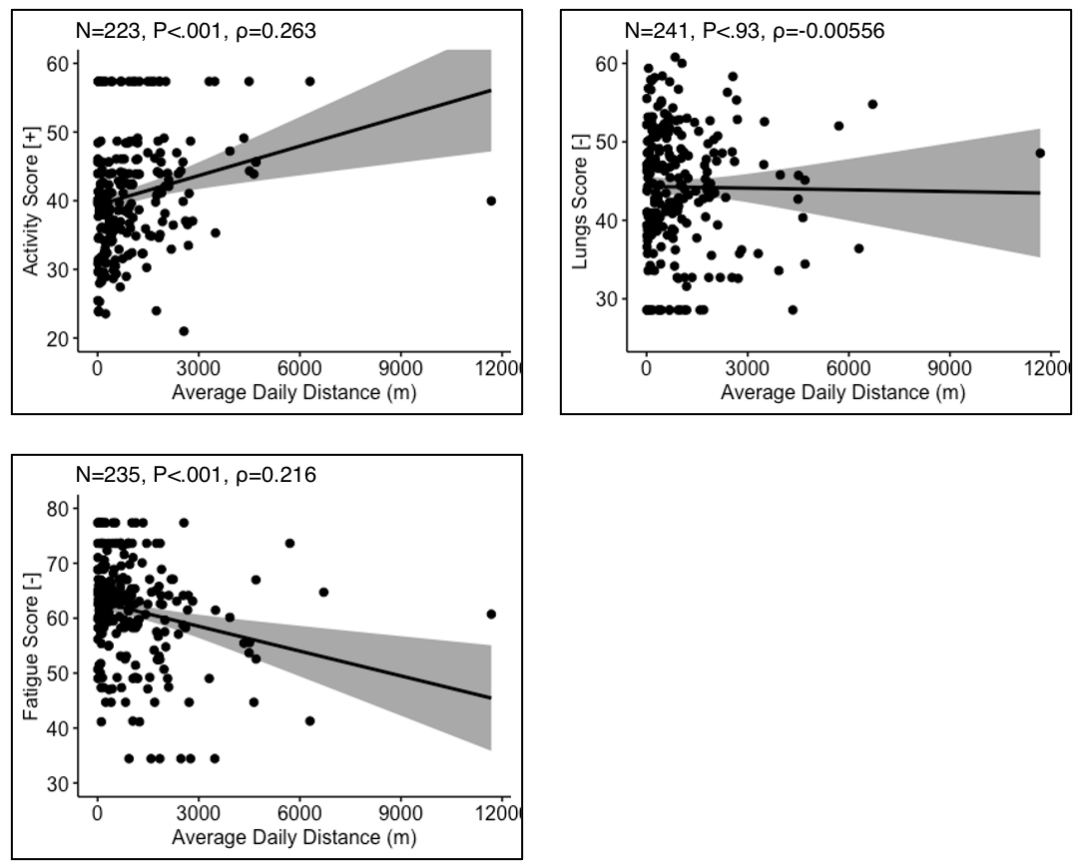
Correlation between Sarcoidosis Assessment Test surveys of physical activity, lung symptoms, and fatigue with device-reported average daily distance traveled. [+] indicates that a higher score represents a higher quality of life. [-] indicates that a higher score represents a lower quality of life. P and ρ are the *P* value and Spearman correlation coefficient, respectively.

Where data were available for both SAT survey responses and device-measured physical activity, correlational analyses were performed. SAT physical activity scores positively correlated with average daily steps (n=226), and SAT fatigue scores negatively correlated with average daily steps (n=245) (Figure 2). SAT lung symptoms scores did not correlate with average daily steps (n=238). These trends were replicated when comparing SAT survey responses to device-tracked daily distance moved (Figure 3).

### Sarcoidosis Medical Resource Usage

Medical resource usage was assessed among 517 unique participants who completed at least one question of an initial survey (Table 3). Overall, 59.8% (n=309) of participants reported at least one regularly scheduled clinic visit in the previous month, while 23.6% (n=122) reported one or more unscheduled clinic visits for sarcoidosis. Furthermore, 11.0% (n=57) of participants reported an emergency room visit for sarcoidosis in the previous month, and 7.4% (n=38) reported a hospitalization related to sarcoidosis. Moreover, 12.4% (n=64) of participants reported missing the entire previous month of school or work because of sarcoidosis, and another 29.4% (n=152) reported missing at least one day of school or work in the previous month.

**Table 3:** Initial response to survey of medical resource usage (N=517).

Variables		Value, n (%)
**How many regularly scheduled clinic visits for your sarcoidosis have you had in the last month?**		
	None	206 (39.8)
	1	153 (29.6)
	More than 1	156 (30.1)
**In the past month, how many unscheduled clinic visits have you had for your sarcoidosis?**		
	None	392 (75.8)
	1	70 (13.5)
	More than 1	52 (10.1)
**In the past month, how many visits to the ER^a^ have you had for your sarcoidosis?**		
	None	442 (85.5)
	1	57 (11.0)
	More than 1	18 (3.5)
**In the past month, how many times have you been hospitalized for your sarcoidosis?**		
	None	478 (94.5)
	1	31 (6.0)
	More than 1	7 (1.4)
**How many days did you miss school/work in the past month because of your sarcoidosis?**		
	None	279 (54.0)
	1 day	45 (8.7)
	2-6 days	73 (14.1)
	7-14 days	22 (4.3)
	>Half month	12 (2.3)
	Entire month	64 (12.4)

^a^ER: emergency room.

### Medication Use and Side Effects

In total, 614 unique participants completed at least one question from the baseline medication survey (Table 4). Overall, 71.2% (437/614) of participants reported using medications to treat their sarcoidosis; of them, 58.8% (257/437) were being treated with prednisone and 81.0% (354/437) were being treated with medications other than prednisone for sarcoidosis. The most common specified medications other than prednisone were methotrexate, hydroxychloroquine, and infliximab.

Participants were also asked to complete a survey concerning medication adverse effects within the prior month (Table 5). Overall, 64.4% (244/379) of participants reported major (requiring changes in medications) or minor side effects from their sarcoidosis medications, with another 20.6% (78/379) of participants reporting possible side effects. The most common medication causing side effects was prednisone. In a follow-up question, participants were most often recommended to continue medications at the same dose when side effects were discussed with physicians.

**Table 4:** Responses to survey of baseline medication usage (N=614).

Variables		Participants, n/N (%)
Are you currently being treated with medications for your sarcoidosis? (Yes)		437/614 (71.2)
Are you taking prednisone for sarcoidosis?		257/437 (41.9)
**Daily prednisone dose (mg)**		
	1-10	131/257 (50.9)
	11-20	65/257 (25.3)
	21-60	57/257 (22.2)
	>61	4/257 (1.6)
Are you taking any medications other than prednisone for sarcoidosis?		354/437 (57.7)
**What other medications are you taking?**		
	Hydroxychloroquine	71/354 (20.1)
	Methotrexate	134/354 (37.9)
	Chloroquine	4/354 (1.1)
	Azathioprine	29/354 (8.2)
	Leflunomide	6/354 (1.7)
	Mycophenolate mofetil	33/354 (9.3)
	Infliximab	51/354 (14.4)
	Adalimumab	28/354 (7.9)
	Other medications	182/354 (51.4)

**Table 5:** Medication adverse effects survey (N=526).

Variables		Participants, n/N (%)
Have you taken any medications for sarcoidosis in the past month?		379/526 (72.1)
**Did you have any side effects from sarcoidosis medications in past month?**		
	Yes, major side effects requiring change in medications	104/379 (27.4)
	Yes, minor side effects	140/379 (36.9)
	Possibly/unsure	78/379 (20.6)
	No	70/379 (18.5)

## Discussion

In this study, we demonstrate the novel use of smartphones to prospectively recruit, consent, and study physical activity and QoL in a large cohort of individuals with sarcoidosis, using both objective health tracking data and qualitative survey responses. We were able to demonstrate a strong correlation between the assessment of physical state and the activity level of participants with sarcoidosis, measured by smartphone apps. Specifically, participants who were more active, as measured by daily steps and distance traveled as tracked by their smartphones, also had physical activity and fatigue scores, representing a smaller impact of their disease on these domains on the SAT—a previously-defined patient-reported QoL metric [7]. Nearly half of the participants missed at least a day of school or work monthly, and nearly one-fifth missed at least a week, reflecting the poor QoL related to physical activity and fatigue reported on the SAT, and demonstrating the profound impact of sarcoidosis on patients. The challenge of pharmaceutical management of sarcoidosis was highlighted by the finding that 6 in 10 participants reported medication side effects, of whom 43% required changes in their medications. Furthermore, 4 in 5 participants reported taking medications for sarcoidosis other than prednisone. Together, these data provide a detailed view into how individuals live with sarcoidosis and demonstrate that smartphones are a compelling method of prospective research for rare diseases, where such wide-scale data collection would otherwise be unfeasible.

The findings of this study suggest that smartphone technology may have advantages in the conduct of prospective clinical research in sarcoidosis and other rare diseases, though robust human and technical resources are critical. First, these data suggest smartphones have potential to enroll patients with sarcoidosis in clinical trials and reliably assess them without requiring a traditional in-person clinic visit. In this way, the clinical research study can be brought directly to participants, bypassing financial and geographic barriers of many socially disadvantaged patients with sarcoidosis, who are often unrepresented in clinical trials. However, these methods cannot replace studies that require laboratory testing or imaging. Second, as these assessments can be made in real time without investigator prompting, this method may avoid significant recall bias and investigator influence respectively. Third, the capability of integrating objective health tracking data with subjective survey data offers a multidimensional assessment of patients with sarcoidosis. In this way, studies using smartphones could provide alternative channels of demonstrating construct validity of patient-reported outcomes.

The strengths of our study were tempered by the technical challenges of developing and maintaining a mobile app. Clinical research teams without strong technical experience will encounter many obstacles in designing and launching mobile apps, and partnerships with technology companies are essential to the success of these projects. However, lack of cross-disciplinary understanding presents substantial challenges to meaningful collaboration with external stakeholders; clinical research teams may not even be able to envision what is technically possible, and developers may lack perspective on how these products are actually delivered to patients [10]. Even with industry partnership, research teams require members skilled in computer science, database management, and data analysis to process the large amount of complex data generated by apps and sensors. For instance, our study lacked longitudinal data collection owing to the challenge of long-term maintenance of the app without dedicated program management or use of participant-engagement rewards systems. Another limitation is that physical activity data could only be tracked if participants were consistently carrying their smartphones, which could not be enforced remotely. As a result, for some participants, physical activity data were sporadically recorded and may not be representative of their actual activity.

There were several limitations regarding the background of participants. First, individuals self-reported a diagnosis of sarcoidosis, which introduces the possibility of participants without a true diagnosis, even though participants were recruited directly from sarcoidosis clinics and advocacy groups for patients with sarcoidosis. Another limitation of our study is that our study population was weighted toward more educated and wealthier individuals. It is possible that the rate of response and familiarity with smartphone apps in our cohort was not representative of the full population of patients with sarcoidosis in the United States, particularly older individuals and those with low technology literacy. Future mobile app studies of sarcoidosis and other rare diseases should prioritize recruiting from a diverse set of sarcoidosis clinics that would provide a more representative sample, in addition to patient advocacy groups. Given that sarcoidosis results in disparate outcomes by race, sex, and socioeconomic class [11] and an annual health care cost of US $20,000 [12], more work is necessary to realize the benefits of the ubiquitous smartphone ownership across all socioeconomic groups.

Future apps could also integrate environmental data, such as location, weather, and air quality to provide additional dimensions of analysis. Wearable devices, such as smartwatches, can also provide valuable data, though ownership is not as prevalent. Beyond observational research, smartphones also present opportunities for digital therapeutics, which are evidence-based interventions driven by software. For example, one group has demonstrated that a smartphone-based stress management tool significantly reduced stress and fatigue in patients with sarcoidosis compared to control patients [13]. In an era of rapid adoption of telehealth driven by the COVID-19 pandemic, clinicians and patients may be more accepting of such tools [14]. The future management of rare chronic diseases such as sarcoidosis may evolve toward using patient-owned devices to actively monitor symptomatology and medication side effects in real time outside of medical centers, allowing rapid treatment adjustment. At the same time, they can also serve as trusted patient education and community platforms, which are highly desired by patients.

## Appendix

Multimedia Appendix 1 Consent quiz to ensure participants understood the risks, benefits, and options of study participation.

## Abbreviations

QoL: quality of life
SAT: Sarcoidosis Assessment Test
STROBE: Strengthening the Reporting of Observational studies in Epidemiology
