# Supplementary material for: Quality of Life and Physical Activity in 629 Individuals With Sarcoidosis: Prospective, Cross-sectional Study Using Smartphones (Sarcoidosis App)
Source: JMIR Mhealth Uhealth. 2022 Aug 10;10(8):e38331. doi: 10.2196/38331 (PMC9403819; doi:10.2196/38331)
Supplement: Multimedia Appendix 1 [file mhealth_v10i8e38331_app1.pdf]

●●●●○ Verizon LTE 1:25 PM 77%

Penn Medicine

Sarcoidosis

Sarcoidosis

A Research Study

Email Consent Document

Swipe to preview

Join Study

Already Participating?

●●●●○ Verizon LTE 1:25 PM 77%

Penn Medicine

Sarcoidosis

About This Study

How can we better understand how sarcodosis and its treatment affects your life?

We want to understand why people with sarcoidosis have different symptoms, why their symptoms vary over time, and what can be done to make those symptoms improve.

The “Sarcoidosis” app will use surveys and phone sensor data to

Join Study

Already Participating?

Penn Medicine

Sarcoidosis

About the App

Please tap on the image below to watch a short video introduction to this app.

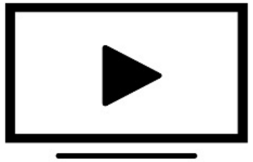

Join Study

Already Participating?

●●●●○ Verizon LTE 1:25 PM 77%

Penn Medicine

Sarcoidosis

How This Study Works

This study will require you to:

1. Register an Account
2. Complete Health Surveys
3. Provide Data

---

1. Register an Account
You need to have the Sarcoidosis app on your phone in order to participate in this study. Each person who enrolls will first complete a consent process explaining the risks and benefits of

Join Study

Already Participating?

●●●●○ Verizon LTE 1:25 PM 77%

Penn Medicine

Sarcoidosis

Who can Participate?

Sarcoidosis is open to people age 18 years or older, who live in the United States, and are comfortable reading English on their iPhone. Data will be collected primarily via the mobile application, so participants must have their own (i.e. not a shared) mobile device / smartphone that can support the application. Participants must be able to provide informed consent and be willing to follow study procedures.

Join Study

Already Participating?

Sarcoidosis

Who's Running This Study?

SPONSOR
The University of Pennsylvania

INVESTIGATOR
Misha Rosenbach

CONTACT INFORMATION
If you have any questions about the study, you can email us at ***. We ask that you do not provide any personal health information over email. However, if you provide us with a phone number, we will be able to contact you to discuss [illegible]

Join Study

Already Participating?

Consent Process

Consent Cancel

Welcome

This simple walkthrough will explain the research study, the impact it may have on your life and will allow you to provide your consent to participate.

Learn more about the study first

Get Started

We'll Test Your Understanding

At the end of this walkthrough, we will ask you a few questions about the details of the study.

Learn more

Next

●●●○○ Verizon 1:58 AM 82%

< Consent Cancel

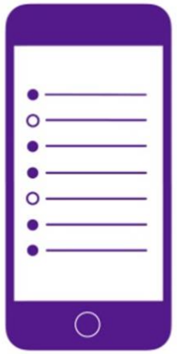

Surveys

We will ask you to rate your symptoms like your energy level and sleep quality daily. We will also ask you to answer brief weekly and monthly surveys about your symptoms to track any changes.

Next

●●●○○ Verizon 1:57 AM 81%

Consent Cancel

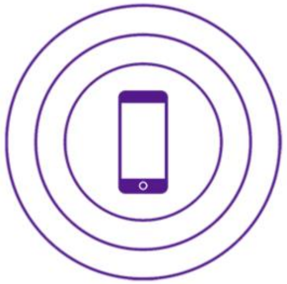

Sensor Data

This study will also gather sensor data from your iPhone and personal devices with your permission.

Learn more

Next

●●●○○ Verizon 1:57 AM 81%

< Consent Cancel

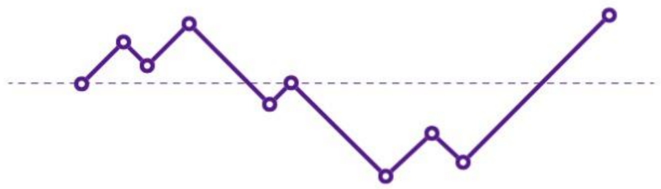

Data Processing

Your study data (survey, tasks and sensors) will be combined with similar data from other participants.

Learn more about how data is gathered

Next

●●●○○ Verizon 1:57 AM 81%

‹ Consent Cancel

Protecting your Data

We will replace your name with a random code. The coded data will be encrypted and stored on a secure Cloud server to prevent improper access.

Learn more about how your privacy and identity are protected

Next

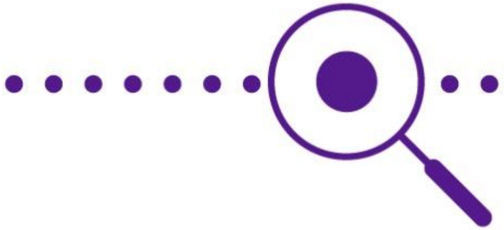

Data Use

The data will be used for this research and may be shared with research partners worldwide.

Learn more about how data is used

Next

●●●○○ Verizon 1:57 AM 82%

Consent Cancel

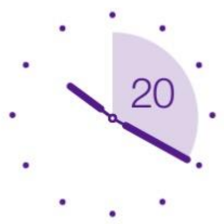

Issues to Consider

This study will take about 15 minutes per week.

Learn more about the study's impact on your time

Next

●●●○○ Verizon 1:58 AM 82%

Consent Cancel

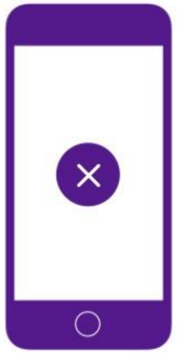

Withdrawing

Your participation is voluntary. You may withdraw your consent and discontinue participation at any time.

Learn more about withdrawing

Next

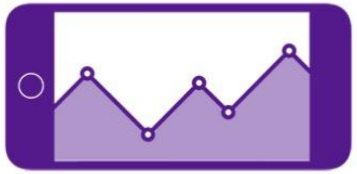

Potential Benefits

You will be able to visualize your data and potentially learn more about trends in your health.

Learn more

Next

●●●○○ Verizon 1:58 AM 82%

‹ Consent Cancel

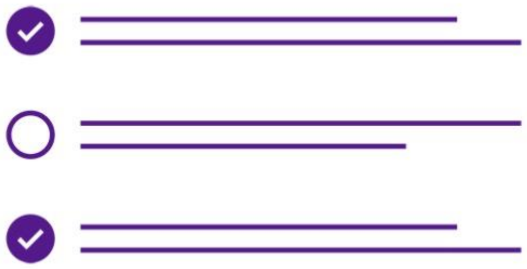

Issues to Consider

This research is not a treatment study. Some questions may make you feel uncomfortable. Simply do not respond.

Learn more

Next

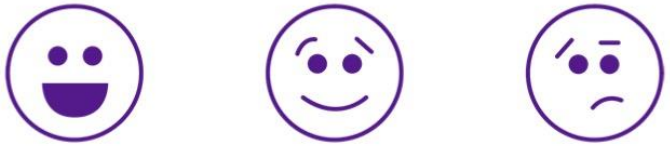

Issues to Consider

Participating in this study could generate a wide range of emotions.

Learn more

Next

●●●○○ Verizon 1:58 AM 82%

< Consent Cancel

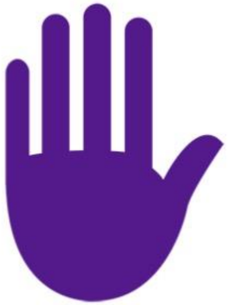

Risk to Privacy

We will make every effort to protect your information, but total anonymity cannot be guaranteed.

Learn more

Next

Sharing Options

Penn Medicine and its partners will receive your study data from your participation in this study.

Sharing your coded study data more broadly (without information such as your name) may benefit this and future research.

Learn more about data sharing

Share my data with Penn Medicine and qualified researchers worldwide

Only share my data with Penn Medicine and its partners

< Consent Cancel

Comprehension

Let's do a quick and simple test of your understanding of this study.

Next

What is the purpose of this study?

Understand how sarcoidosis affects quality of life.

Treat sarcoidosis symptoms

●●●○○ Verizon 2:15 AM 83%

Consent Cancel

My name will be stored with my study data.

Yes

No

I can choose to share my coded study data sparsely or more broadly.

Yes

No

Consent Cancel

I will be able to skip any survey question.

Yes

No

I will be able to stop participating at any time.

Yes

No

●●●○○ Verizon 2:15 AM 83%

Consent Cancel

Great Job!

You answered all of the questions correctly.
Tap Next to continue.

Next

●●●○○ Verizon 2:15 AM 83%

Consent Cancel

Review

Review the form below, and tap Agree if you're ready to continue.

Consent

STUDY INFORMATION AND CONSENT to RESEARCH

TITLE: Sarcoidosis (A research Study)

PROTOCOL NO.:***

SPONSOR: Penn Medicine

INVESTIGATOR:

Misha Rosenbach MD

Disagree Agree

●●●○○ Verizon 2:15 AM 83%

Consent Cancel

Review

Review the form below, and tap Agree if you're ready to continue.

Con[illegible]

STU[illegible] **T to**

TITL[illegible]

> **Review**
>
> By agreeing you confirm that you read the information and that you wish to take part in this research study.
>
> Cancel | Agree

PROTOCOL NO.:***

SPONSOR: Penn Medicine

INVESTIGATOR:

Misha Rosenbach MD

Disagree Agree

Consent

First Name Required

Last Name Required

Next

Consent Cancel

Signature

Please sign using your finger on the line below.

Sign Here

Done

Registration

Cancel Consent

What to Expect

On the next screen, you will be prompted to grant Sarcoidosis access to read and write some of your general and health information, such as height, weight and steps taken so you don't have to enter it again.

Got It

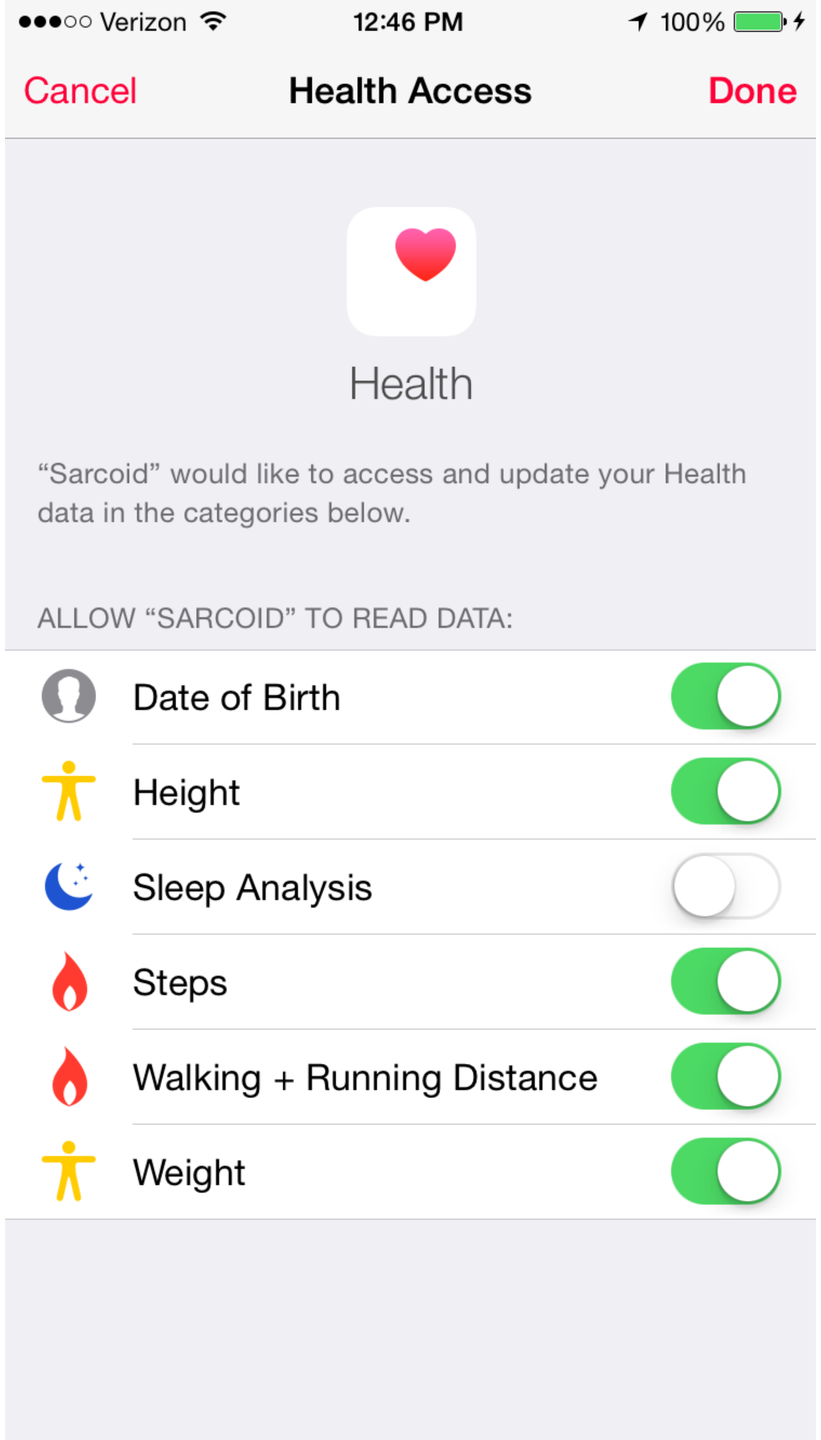

Verizon
12:46 PM
100%
Cancel
Health Access
Done
Health
“Sarcoid” would like to access and update your Health data in the categories below.
ALLOW “SARCOID” TO READ DATA:
Date of Birth
Height
Sleep Analysis
Steps
Walking + Running Distance
Weight

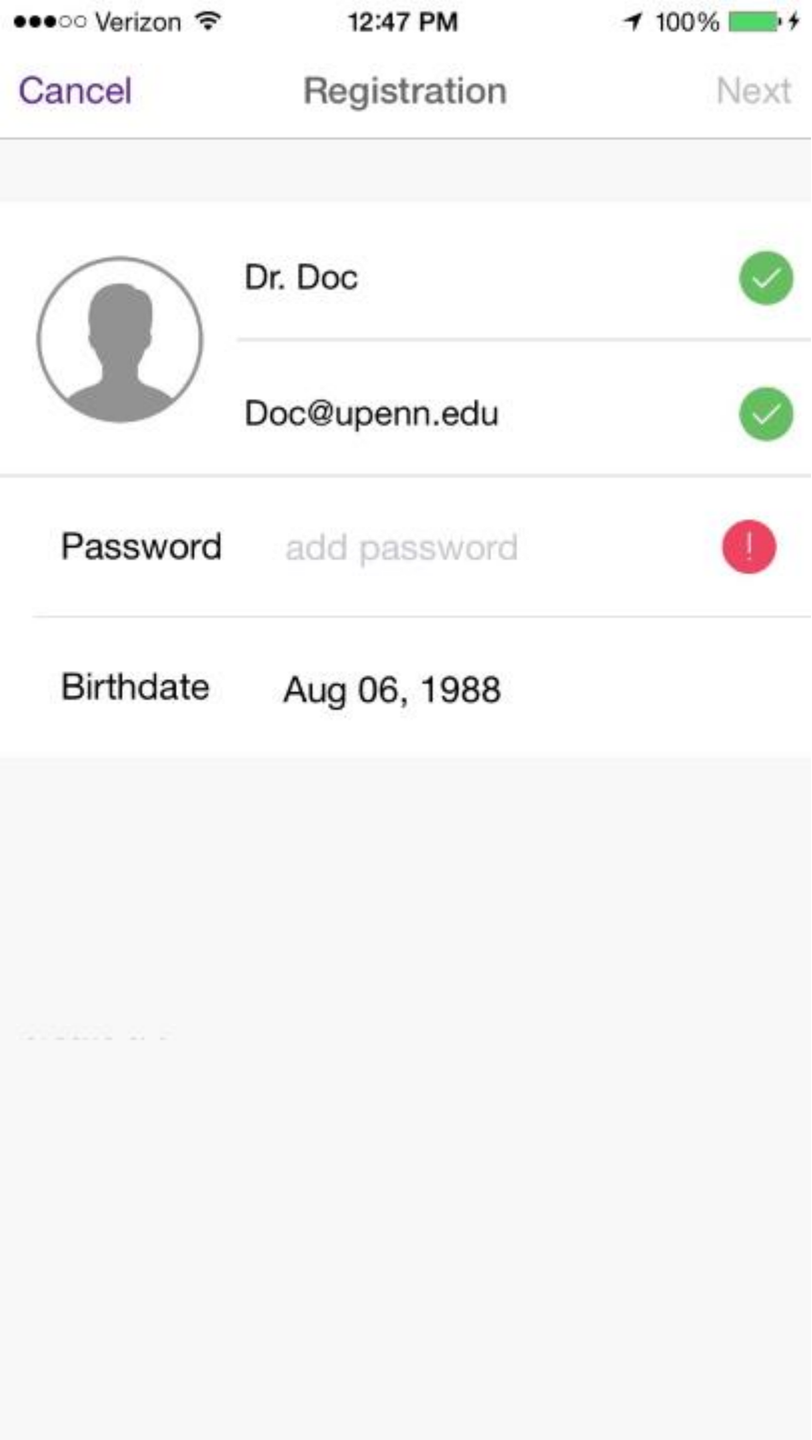
Verizon
12:47 PM
100%
Cancel
Registration
Next
Dr. Doc
Doc@upenn.edu
Password
add password
Birthdate
Aug 06, 1988

●●●○○ Verizon 12:47 PM 100%

Additional Information | Next

Height	6' 1''
Weight	180

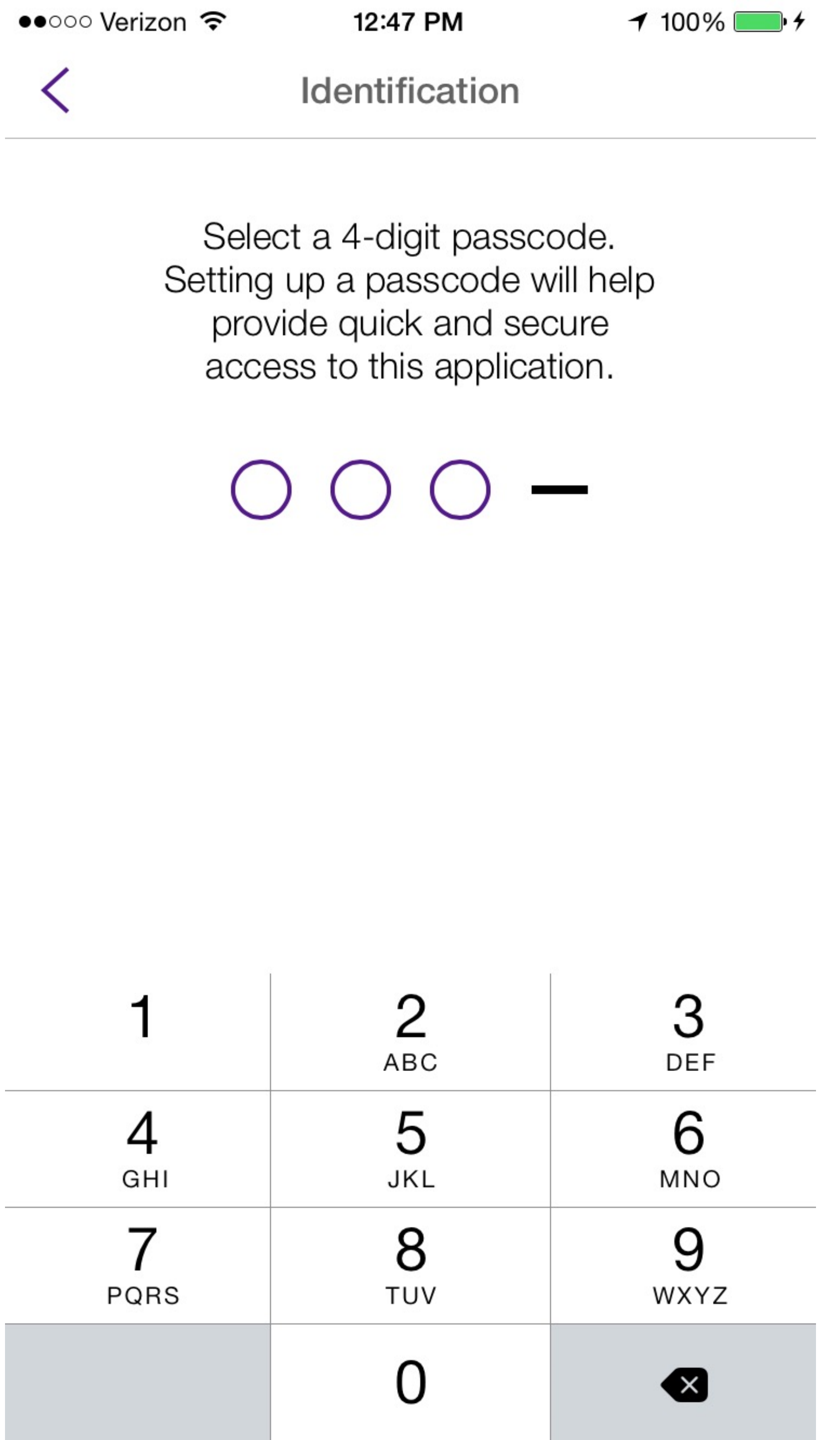
Verizon
12:47 PM
100%
Identification
Select a 4-digit passcode.
Setting up a passcode will help
provide quick and secure
access to this application.
1
2
ABC
3
DEF
4
GHI
5
JKL
6
MNO
7
PQRS
8
TUV
9
WXYZ
0

●●○○○ Verizon 12:47 PM 100%

Permissions Done

Location Services

Using your GPS enables the app to accurately determine distances travelled. Your actual location will never be shared.

Allow

Motion Activity

Using the motion co-processor allows the app to determine your activity, helping the study better understand how activity level may influence disease.

Allow

Notifications

Allowing notifications enables the app to show you reminders.

Granted

●●○○○ Verizon 12:47 PM 100%

Email Verification

Sarcoidosis
has sent you
a verification email at

Doc@upenn.edu

Wrong email address? Tap here.

Once verified, tap below

Continue

A full copy of the consent agreement
will be sent to you for your records.

Resend Verification Email

●●○○○ Verizon 12:48 PM 100%

All Set!

Thank you for enrolling in

Sarcoidosis

This is your app to use as you choose.

You'll find your list of daily surveys and tasks on the "Activities" tab. New surveys and tasks will appear over the next few weeks.

You will be able to log, as often as you like, your energy, sleep, excercise, breathing and an activity of your choice.

To see your results from surveys and tasks, check your "Dashboard" tab.

Let's Begin
